# Unravelling the spirits’ message: a study of help-seeking steps and explanatory models among patients suffering from spirit possession in Uganda

**DOI:** 10.1186/1752-4458-8-24

**Published:** 2014-06-09

**Authors:** Marjolein van Duijl, Wim Kleijn, Joop de Jong

**Affiliations:** 1 Netherlands Institute for Forensic Psychiatry, The Hague, The Netherlands; 2 Leiden University Medical Center, Leiden, The Netherlands; 3 Centrum '45, Oegstgeest, The Netherlands; 4 Amsterdam Institute for Social Science Research, University of Amsterdam, Amsterdam, The Netherlands; 5 Boston University School of Medicine, Boston, USA

**Keywords:** Spirit possession, Uganda, Explanatory models, Help-seeking, Dissociative disorders, Traditional healing, Traumatic experiences

## Abstract

As in many cultures, also in Uganda spirit possession is a common idiom of distress associated with traumatic experiences. In the DSM-IV and -5, possession trance disorders can be classified as dissociative disorders. Dissociation in Western countries is associated with complicated, time-consuming and costly therapies. Patients with spirit possession in SW Uganda, however, often report partial or full recovery after treatment by traditional healers.

The aim of this study is to explore how the development of symptoms concomitant help-seeking steps, and explanatory models (EM) eventually contributed to healing of patients with spirit possession in SW Uganda. Illness narratives of 119 patients with spirit possession referred by traditional healers were analysed using a mixed-method approach.

Treatments of two-thirds of the patients were unsuccessful when first seeking help in the medical sector. Their initially physical symptoms subsequently developed into dissociative possession symptoms. After an average of two help-seeking steps, patients reached a healing place where 99% of them found satisfactory EM and effective healing. During healing sessions, possessing agents were summoned to identify themselves and underlying problems were addressed. Often-mentioned explanations were the following: neglect of rituals and of responsibilities towards relatives and inheritance, the call to become a healer, witchcraft, grief, and land conflicts.

The results demonstrate that traditional healing processes of spirit possession can play a role in restoring connections with the supra-, inter-, intra-, and extra-human worlds. It does not always seem necessary to address individual traumatic experiences *per se,* which is in line with other research in this field. The study leads to additional perspectives on treatment of trauma-related dissociation in Western countries and on developing effective mental health services in low -and middle-income countries.

## Background

### Attribution and treatment of dissociative disorders in Western countries

In DSM-5, possessive trance disorders are included under dissociative identity disorder (DID), owing to phenomenological similarities [1,2]. There is strong empirical support for the hypothesis that dissociation is caused by traumatic stress and that dissociation is related to a history of trauma even after controlling for fantasy proneness [3]. The trauma theory of dissociation is supported by research on dissociation as a regulatory response to fear or other extreme emotions with measurable biological correlates [4]. In the World Mental Health Survey conducted in 16 countries, dissociative symptoms (such as depersonalization and derealization) were present in 14.4% of the respondents with PTSD. This did not differ in high/middle and low-income countries. High dissociation was associated with more severe PTSD, high role impairment and suicidality, a history of early onset, and a history of multiple childhood trauma adversities [5]. Severe dissociative disorders contribute to functional impairment above and beyond the impact of non-dissociative Axis I disorders and, as such, qualify as serious mental illness [6]. Despite the relationship with trauma, standardized PTSD treatment is only partially effective for severe dissociative symptoms [7].

### Attribution and treatment of dissociative disorders in African countries

In  non-Western’, low-resource settings, spirit possession as a dissociative phenomenon is a common attribution and expression of distress. Although anthropologists have provided numerous examples over the recent decades [8,9], the last few years have shown a growing attention in medical–psychiatric literature to the relationship between psychological distress, traumatic events, and spirit possession [10-17]. In an epidemiological study in a population of 941 adults in post-war Mozambique, Igreja et al. found a prevalence of 18.6% adults suffering from at least one spirit [12]. Neuner et al. found that 8% of a population of 1,113 youths and young adults in Northern Uganda suffered from severe forms of spirit possession [14].

All respondents (medical workers, counsellors, traditional healers, and religious leaders) in a qualitative study in SW Uganda confirmed the common presence of dissociative trance and possessive trance states, manifesting as possession by *omuzimu *(ancestral spirits)*, emandwa *(messenger spirits) and *bachwezi* (demi-gods). The possessing agents were said to manifest themselves to address neglected rituals and unresolved conflicts [18]. In a subsequent case-control study in SW Uganda, patients (n = 119) with spirit possession in comparison with controls reported significantly higher levels of locally recognized dissociative symptoms, more severe psychoform dissociation and somatoform dissociation on scales developed in the West, as well as more potentially traumatizing events [13]. The correlations between traumatic events and different types of dissociation were very high. Traumatic experiences, however, were not spontaneously mentioned prior to or during the treatment by healers, nor were they specifically addressed during the healing process. This makes it interesting to look in more detail at how these patients with spirit possession, who according to Western measures suffered from severe trauma-related dissociative symptoms, evaluated the local treatment process. Which help-seeking steps did they undertake, and how were local explanatory models (EM) generated in the search for healing? How did they experience the final outcome?

### Pathways to healing

Help-seeking behaviour for a health problem can be defined as problem-focused, planned behaviour, involving interpersonal interaction with a selected health care professional [19]. Explanatory models (EM) is a term introduced by Kleinman, referring to “notions about an episode of sickness and its treatment that are employed by all engaged in the clinical process” [20]. Recent studies in Africa on EM—concerning various common mental disorders such as psychosis [21-24], epilepsy [25], depression [26], or mental illness in general [27-30]—illustrate a wide range of locally used categories and explanations for Western categories of mental illness. It is also acknowledged that traditional healers play an important role in dealing with distress, especially in countries where Western psychological services are extremely limited [31,32]. In addition,  pathway to care’ studies in 11 countries [33] demonstrated that native healers often play an important role in addressing mental health problems in low-resource settings. Despite increasing attention to the role of traditional healers and cultural explanations in relation to various mental disorders, attention to dissociative disorders as a specific category of mental disorders is still limited in psychiatric research in the African context.

To compare trauma processing mechanisms across the globe, De Jong and Reis propose analysing these mechanisms with the help of a comprehensive model with five universal ontological dimensions involved in suffering and healing [15]. These dimensions are defined as (a) intra-human (mind–body); (b) inter-human (social interactions); (c) supra-human (ancestors, gods, embodied entities); (d) extra-human (ecology, nature, cosmos, animals); and (e) time (relationship between past, present and future). They applied this model to describe how a dissociative cult in Guinea Bissau, the Kiyang-yang, provided an idiom of distress in the context of political violence and enabled collective trauma processing.

The aforementioned epidemiological studies demonstrate that spirit possession in different African contexts can be associated with severe dissociative symptoms and impaired social functioning. Similar to research results in Western countries, dissociative disorders are related to severe traumatic experiences [12-14].

In the theory of trauma-related structural dissociation, dissociation is described as an integrative failure manifesting in positive and negative symptoms or personality parts [34,35]. This fits with our findings in Uganda described in a former publication on the symptoms of spirit possession compared with the DSM-IV and 5 [36]. Multiple correspondence analysis of dissociative symptoms of patients suffering from spirit possession revealed dimensions of symptoms that could be characterized as positive and negative or active and passive. We suggested that spirit possession can be an idiom of distress in a context of suppression and societal disruption and that it is comparable to the role of an “emotional part of the personality” containing conflicting and traumatic experiences that are incompatible with the expected normal behaviour in a specific traditional, political, or religious context.

Whereas the current standard in Western societies for treatment of dissociative disorders is phased trauma-focused therapy [4,35,37], it is often a long, costly process with limited effectiveness [4,38]. In African contexts, severe dissociative symptoms are often seen as an expression of spirit possession and dealt with by traditional healers. It is therefore useful to gain more insight into the process of help-seeking and the generation of explanations that lead to treatment in the African context.

### Aim

The aim of this study is to explore the pathways to healing of patients with spirit possession who visit traditional healers in SW Uganda, by exploring their help-seeking behaviour, the healing methods used by the healers, the EMs that endorsed the healing process, and the perceived subjective effectiveness of the healing process.

## Methods

### Context of the study

The study area comprises three districts—Mbarara, Bushenyi, and Ntungamo—in SW Uganda, with a total population of approximately 1.5 million inhabitants. The majority of people live in rural areas, and the economy depends mainly on agriculture, mostly on a subsistence level.

### Participants and procedure

The participants of this study consisted of the case group of 119 patients with spirit possession of the aforementioned case-control study [13]. The procedures of the case-control study have been described in former studies [13,36]; relevant steps for the current research question are summarized here. With the help of key informants, inventory lists were made of approximately 300 traditional healers. Those who reported to offer treatment for spirit possession (80 healers in 19 healing places) were approached and asked to refer patients suffering from possession by a spirit or power, or as the result of witchcraft or sorcery by another person. Referred patients (n = 120) were interviewed at the healer’s place on our next visit. It was explained to the interviewed patients that we wanted to learn more about spirit possession. A Ugandan research assistant, a student in Development Studies, conducted the interviews in the local language (Runyankore) under supervision of the first author. One patient was excluded because of incomplete information. The group of 119 patients with spirit possession consisted of 45% male and 55% female patients. The mean age in the case group was 38.4 years (SD = 12.2). Approximately 23% had no education, and around 60% had stopped schooling during or after primary school.

All participants gave verbal informed consent prior to inclusion in the study, and procedures were in accordance with the Helsinki Declaration [39]. Study and procedures were approved by the International Research Review Board of Mbarara University and the District Authorities in Uganda.

### Instruments

The Spirit Possession Questionnaire-Uganda (SPQ-Ug) included open-ended questions enquiring about a participant’s history of symptoms and help-seeking steps, the healing place and healing methods used, the result of treatment, and the subjective explanation of their possession. These open-ended questions resulted in mini illness narratives on the subjective experienced symptoms of patients with spirit possession, and on the above mentioned topics. The questionnaire was translated into Runyankore and administered verbally by the local research assistant because of the high illiteracy rates among the participants of the study. The narratives were recorded in English by the interviewer.

The SPQ-Ug was an interview checklist locally designed by the first author with support of the mental health team of the psychiatry department in Mbarara. The first part listed demographic items such as name, age, sex, home address, healing place, religion, education level, occupation, marital status, and number of children (living and deceased). It continued with a checklist of open questions on the history of the problem (how did it start, with which symptoms, what preceded the possessive state); how the possessive state was characterized (such as noises, body movements, words) and which treatments had been tried; how the patient and others explained the symptoms; the psychosocial events (stressful events, family conflicts, cultural conflicts such as concerning dowries, rituals); which healing methods were used, how did the patient learn about it; how did the patient experience the healing and what further expectations did they have. The interview can be characterized as semi-structured and problem-centered, with open questions to provide room for the patients’ narratives and involving their personal descriptions and explanations [40].

### Data analysis

A mixed-methods research design was used to examine the following topics:

A. Symptoms, help-seeking steps, and referral

B. Healing type at the final healing place

C. Healing procedure, possessing agents, and outcome of treatment

D. Explanatory models

Data analysis was performed by the first author and supervised by the second and third authors. A combination of strategies was used for content analysis. Most categories for coding were directly guided by the open questions of the interview (e.g. type of healer, explanation, help-seeking steps, method of healing, and evaluation of treatment). These can be defined as  analytical units’, according to Flick [40]. On large overhead sheets a handwritten overview was made with paraphrased passages of the narratives covering these analytical units for all 119 patients. The next level of themes was distilled from the text and was added in the overview—for example, the types of spirits that were involved. Types of explanatory models (EMs) were based on the themes in the explanations that were applicable for the final healing step. Defining these EMs is a process of  conceptual ordering’ that requires a certain level of abstraction [41]. These were often mentioned explicitly by the patient in the narrative (e.g. neglect of rituals), but they could also be more implicit. The EMs were coded and entered in the handwritten overview. Abstraction in conceptual ordering involves the judgment of the researcher and can be biased. To crosscheck validity, types of spirits and explanatory models were discussed with traditional healers, medical students, and colleagues in the psychiatry department in Mbarara on several occasions. Results were entered into SPSS, which was used for basic statistics such as percentages and exploring relationships with cross-tables. Final results of the analysis presented in this paper were evaluated with the local interviewer.

## Results

The following sections will sequentially address the topics listed above (A–D). Typical examples in the narratives were selected to illustrate these topics.

### Symptoms, help-seeking steps and referral

The history of the patients with spirit possession typically began with physical problems or other misfortunes. Common physical symptoms were fever, headaches, body pains, vomiting, a swollen stomach or ulcers. Other patients with spirit possession emphasized misfortunes such as many children that had died, infertility, or even a stolen bicycle.

Gradually, in the course of weeks or months, the patients develop dissociative and sensory-motor symptoms, described as fainting, seeing through fog, not being able to move or speak, and seizures. Often these symptoms begin or become stronger after one or more steps in the professional health care sector are unsuccessful. Subsequently, patients mention passive-influence experiences of spirit possession, such as feeling held or influenced by powers from outside, strange dreams, hearing imperative voices, or attacks of shaking movements, or they may begin to speak in another language.

The following case illustrates some of these dissociative experiences:

Id 43. Female: age 40

It was three years ago that I started having the following complaints. At first, I sort of lost sight—as if I was looking at the world through fog. Objects would seem either small or too big. I was forgetful of what I intended to do; I would just sit there without doing anything. I would hear some voices in my head directing me what to do. Some big man would also come to me almost every night in a dream, and he would threaten to kill me. My husband tried his best to take me to several health centres. They gave me some tablets and a few injections—but in vain.

During this process, spiritual causes were considered, and referral to traditional healers and occasionally church healing was suggested by the environment of the patient. Passive influence experiences of spirits were followed by the actual active state of spirit possession, in which the spirit presents itself through the patient, characterized by changes in consciousness, shaking movements, and talking in a voice attributed to spirits. In half of the cases, the active presentation of the spirits occurred before the healing ritual; in the other half, these possessing agents began to appear and speak through the patient only during the healing session.

Table 1 shows that 66% of the patients first sought help from  Western’ medical services such as hospitals (37.8%) and health centres (16.8%), or tried self-prescribed medicine (11.8%). Only 26% of our case group directly consulted traditional healers, and 8% tried healing through the church as a first step.

**Table 1:** Initial choice of treatment mode

**First step (initial choice of treatment mode)**		
**Treatment mode**	**n**	**%**
**Medical**		
*Medicines/self-help*	14	11.8
*Health centre*	20	16.8
*Hospital*	45	37.8
**TOTAL**	**79**	**66.4%**
**Traditional healers**		
*Omuraguzi (original tradition)*	24	20.2
*Barangi (Christian elements)*	7	5.9
**TOTAL**	**31**	**26.1%**
**Church-related treatment**		
**TOTAL**	**9**	**7.6%**
**TOTAL patients**	119	100%

All patients with the first help-seeking step in the medical sector and a few from the traditional healers (n = 2) and churches (n = 4) did not find relief. These patients (in total, 70% of all patients) subsequently undertook a second help-seeking step: 28% of the patients in the medical sector, 37% visited traditional healers, and 5% went to a church healing location. After the third step, undertaken by a third of the patients, most of the patients had reached their final healing place. On average, patients with spirit possession tried two steps in help-seeking before reaching the healing place where they achieved positive results.

More than half (58%) of all patients with spirit possession were referred by their relatives (parents, siblings, uncle); a quarter (25%) by their neighbour; and 5% by friends. A further 12% did not answer this question.

### Healing type at the final healing place

The final healing places can be categorized into three major types. The majority (54%) were conventional traditional healers called *omufumu*. These healers usually inherit their healing powers through their patrilineage or matrilineage and act according to traditional cultural practices. The second type were the *barangi* healers (30%), who use a mixed approach of traditional healing and Christian elements. They work and live together in a group that can be described as a therapeutic community and is based on  the Ten Commandments of the Bible’. Their patients often come from afar and stay in the community of these healers for some time, often weeks, and participate in the agricultural work of the community while joining in healing sessions. A relatively small group (16%) of the patients in our study was referred by the third type of healing places, i.e. church healing places using the healing force of the  Holy Spirit’. These were referred to as the Miracle Church, the Holy Spirit Church, and Pentecostal and Charismatic churches.

### Healing procedure, possessing agents, and outcome of treatment

Most patients described how, at the healer’s place, the possessive agent was invited to manifest itself through praying, singing and dancing. Christian prayers and songs were used by *barangi* healers and in church healing sessions. Sometimes the process was supported by giving herbal medicine to the patients (orally or by rubbing herbs into cuts on the body). Before healing, a patient could involuntarily experience spirits through dreams, hearing voices, and having sensory-motor experiences; now these spirits eventually manifested themselves through the patient while he/she was in a full dissociative state, and the spirits could start to communicate. Through the communication by the spirits, underlying problems became clear.

The possessing agents were referred to as *bachwezi* (demi-gods) by 26% of the possessed patients, and as *omuzimu* (ancestral spirits) in 49% of the cases, such as the spirit of a deceased grandmother, father or brother. Of the 119 cases, 25% suffered from witchcraft and sorcery. In the last group, the possessing agents were referred to as *amahembe* (bad spirits or malevolent forces) or the spirits of a living jealous person (e.g. a neighbour or co-wife). Witchcraft and jealousy allegations played a more prominent role at the *barangi* healers (44%) than at the *omufumu* healers (17%) and in church healing settings (16%). Ancestral spirits were significantly less involved among patients of *barangi* healers (25%) than among those at *omufumu* healing settings (59%) and church settings (58%). The occurrence of *bachwezi* spirits was almost similar in these different settings.

The following is an example of the voice of a *bachwezi,* reminding the afflicted person of her spiritual lineage and obligations:

Id 33. Female: age 50

This healer gave me medicine to drink and then started praying for me. It started by simple nodding of my head. Then my whole body followed. The voice said: “She is our child; she has to come back.”

The following example demonstrates how an ancestral spirit (a deceased father*, omuzimu*) reminds his son that he has neglected his responsibilities of taking care of his mother:

Id 86. Male: age 52

… Then I was brought here just a week ago. The healer made me smell cock feathers; he even cut my body and rubbed some medicine within. Then the voice of my late father came out, saying: “I am angry with you, my son—why did you chase away your mother? Why should your wife influence you and disfavour your real mother, my wife?” This spirit of the father directed me to bring my mother back home. This healer has since then given me a drinking medicine. I am now getting healed. This healer has helped me greatly. I hope to go home soon.

Often the healer negotiated with the spirits about forgiveness of past evil deeds or negligence of ritual, and sometimes protection against the recurring possession episodes was provided by herbs administered orally or via cuts in the body. Regularly, the patients promised to change their behaviour. Some patients were called by the spirits to be initiated as a healer. Many acknowledged and revived worship of the ancestral powers in the family, which they had neglected before. Concerning the evaluation of their treatment the patients were asked how they had experienced the treatment and what they expected as a result. At the time of the interview when patients evaluated their treatment, 42% patients felt much better and relieved, and 57% reported being completely healed. An example of a patient feeling much better is a woman who said: “voices have stopped disturbing me”; and she described her expectation: “I will be alright and do my daily work without any problems”. Another patient who felt completely healed said: “It [the Amahembe, spirits that wanted to kill the patient as a punishment for her father’s behaviour over land conflicts with his neighbour] has never come back. I hope to go back to my husband and to go on with life as before”. Only two had doubts and feared their problems might return. Complete healing was reported by relatively more patients of the *omufumu*, the conventional traditional healers (66%), compared with church (55%) and *barangi* healing (30%) settings.

### Explanatory models

The types of possessing agents and the types of messages they conveyed (EM) are listed in Table 2.

**Table 2:** Explanatory models and types of possessing spirits

	**Explanatory model**															
	**Ritual neglect**		**Neglected responsibilities**		**Jealousy**		**Call to become a healer**		**Land conflict**		**Death of dear one**		**Spirits no clear reason**		**TOTAL type of spirit**	
**Type of spirit**	**n**	**%**	**n**	**%**	**n**	**%**	**n**	**%**	**n**	**%**	**n**	**%**	**n**	**%**	**n**	**%**
***Bachwezi *** **(demi-gods)**	20	59%	-	-	-	-	11	92%	-	-	-	-	-	-	**31**	**26%**
***Omuzimu *** **(ancestral spirits)**	13	38%	22	73%	-	-	1	8%	8	67%	11	100%	3	50%	**58**	**49%**
**Witchcraft**^**a**^	1	3%	8	27%	14	100%	-	-	4	33%	-	-	3	50%	**30**	**25%**
**TOTAL expl. model**	**34**	**29%**	**30**	**25%**	**14**	**12%**	**12**	**10%**	**12**	**10%**	**11**	**9%**	**6**	**5%**	**119**	**100%**

^a^Witchcraft, e.g. initiated by neighbour or co-wife.

Note: Chi-square (12, 119) = 120.28, p < .001.

The *bachwezi* (demi-gods) were related to ritual neglect and the call to become a healer. Ancestral spirits were most often related to neglected responsibilities, such as looking after the inheritance of a relative who had died, his family, land, and cows. But ritual neglect, land conflicts, or death of a dear one can also be connected themes. Witchcraft is most often related to themes of jealousy and can also be associated with neglected responsibilities and land conflicts. Explanations were not dependent on sex, age, or education level.

EMs related to neglect of rituals and responsibilities, communicated by the *bachwezi* and ancestral spirits, were relatively more associated with *omufumu* traditional healers and the experience of  complete healing’; and EMs related to jealousy, grief, or spirits, and without a specific message, were slightly more present at the *barangi* healers and were more often related to feeling “a lot better” after treatment.

It should be noted that in the preceding process of development of symptoms and the various help-seeking steps, different and sometimes conflicting EMs can be considered by the patient and his relatives. For example: owing to initial medical symptoms, biomedical EMs may have been considered, such as malaria or HIV symptoms. Even when spiritual causes might be considered, patients might initially refrain from seeking help from traditional healers, as this might not be in accordance with their Christian or Islamic religion. In the final healing place the following groups of EM were found in rank order from most to least common:

(i) ritual neglect (ritual being a precondition for connection to the spiritual world)

(ii) neglect of responsibilities (e.g. concerning care for the inheritance or family after the death of a father/mother/brother)

(iii) jealousy (e.g. by co-wives or neighbours)

(iv) the call to become a healer

(v) grief

(vi) land conflicts

(vii) others.

The content of these EMs will be further illustrated below.

In some cases, a patient’s narrative represented a combination of interconnected EMs. For instance,  ritual neglect’ could be associated with  land conflicts’ (e.g. when land where ancestors were buried had been sold and rituals could no longer be performed there).

Specific sources of distress or specific physical symptoms could be attributed to different EMs by different individuals. For example, a common source of distress such as barrenness or infant mortality could be associated with EMs such as  the call to become a healer’,  jealousy of a co-wife’, or  unpaid bridal prices’. For example, one man (Id. 69) lost six children before they were three years old. In the healing session, the *bachwezi* ordered him to become a healer. Now he has four children.

(i) Ritual neglect

The most commonly perceived cause was angry and disappointed *bachwezi* (demi-gods) and other spirits because of ritual neglect (N = 34, 29%). These rituals were important for reinforcing the connection with the spiritual world, such as the spirits of the ancestors and the *bachwezi,* who guard the lineage of the clan and family. Under pressure of the church, these traditional rituals were often discontinued; and items used for these rituals, often kept in a small shrine in the homestead, were burnt^a^. Ritual neglect is illustrated in the following example:

Id 6. Male: age 26

*Possession began on a hospital bed, where late in the night I uttered words saying “We want houses” and words like “You abandoned us”. These voices were spirits of our ancestors. Actually, it was a combination of* omuzimu *and* emandwa^b^, *who were seeking houses. They wanted to be brought back after being abandoned. They wanted a goat to be slaughtered for them, and I should take the blood of this goat to welcome the spirits back. My father, who used to practise rituals for ancestors, has died. I have ignored these rituals. The spirits then decided to manifest themselves through me.*

The treatment provided by this healer has helped me, whereby some medicine was put in my body and I have some which I still take regularly. I feel relieved and am grateful. I have experienced a lot of relief and feel far better. I expect to heal completely.

(ii) Neglect of responsibilities

In 31 cases (26%), the patient experienced possession as a punishment because of neglecting responsibilities or because of misbehaviour, even to the extent of untruthfully acting as a healer.

These are usually patients suffering from the spirit of a deceased relative, who came to punish the patient for not having taken proper care of the deceased’s inheritance. After the death of a father or brother, the son or remaining brother becomes the heir and is responsible for looking after the property, land (agriculturalists) or cows (in the case of cattle herders), and the widow(s) and children of the deceased. This is illustrated in the following example:

Example of family conflicts, not managing responsibility well, church solution: Id 44. Male: age 40

After the death of my brother, I was given the responsibility of looking after his widow and children. My brother had a big farm and a vehicle, so I started using the vehicle as a taxi. I would take some little money every day to the widow, and I managed to build myself a house profiting from this vehicle. We used to sell off cows in the farm whenever we wanted school fees for the orphans. It seems the widow used to complain that I was misusing her vehicle, while giving her too little money. And indeed, that was the truth. So the spirit of my brother came to punish me. His spirit came once during the day, while we were having lunch, and he knocked me down. He started speaking out through my mouth and asking why I was making his wife and children suffer. Fortunately, it was on a Sunday and my sister rushed to this church and requested the brethren, who came, prayed for me and rescued me.

Some of the patients felt punished for not treating well their husband (e.g. after separation), sister-in-law, or (co-)wife. The spirit of a deceased husband expressed anger with his wife for seeing another man after his death. In other cases, the spirits of the deceased did not agree when land (belonging to the family) was being sold. One woman was punished because she did not marry a Muslim man.

(iii) Jealousy

Anthropological literature distinguishes between envy and jealousy [42]. Envy is characterized by feelings of inferiority, longing, resentment, and disapproval of the emotion. Jealousy is characterized by fear of loss, distrust, anxiety, and anger. Locally the term jealousy was used for both situations. Jealousy played a role in 14 cases (12%). This could be bewitchment—for example, by a neighbour, because of owning more cows or having a better roof on the house; by a co-wife, because of having more sex or privileges; or by a co-worker in the market, because of doing better in business.

Example of jealous wife: Id 25. Male: age 50

I had married two wives. They lived in separate but nearby homesteads. The young one did not feel good whenever I visited the first wife for a night. For this reason, she decided to give me some medicine which she got from a witch doctor, which was supposed to stop me from visiting the first wife. It looks like she did not follow the right directions according to the witch doctor, and the herbs made me go mad. They took me to the hospital, but the situation worsened until they brought me here.

The possessive state was characterized by noises and a lot of shaking when they prayed for me. Some voice would speak out: “What don’t I have that makes you go to another woman? I want you to stay with me alone”.

(iv) Call to become a healer

Twelve patients (10%) eventually learned that they were predestined to become a healer themselves. Some of them had dreams about a white person taking them somewhere or about herds of cattle^c^. Some of them had been speaking in unknown languages.

Example of becoming a healer: Id 100. Male: age 40

The possessive state started during the healing sessions.

I developed a sore as a small boy and was taken to several health centres, but the thing would not dry up. It pained me a lot and it prevented me sleeping. It was disturbing me a lot. We had a Charismatic church nearby, and its people claimed that they would pray for me and I would get healed. They tried but the sore remained. One night a voice came and told me that my healing was only in Biharwe, behind the trading centre. It even told me that Mrs. K. was the healer and she would pray for me and get me healed. When I told my parents in the morning, they decided to bring me there. This healer made me sit on the mat with other sick people and prayed aloud. The voice at once came out of me, saying: “You are now at home; your healing place is here; feel free, my son; you have to help many other suffering people like you”. Within two weeks, the sore had dried out and I got healed. That was some years back. I now have a healing centre at home.

Example of becoming a healer: Id 101. Female: age 53

*My health problem developed when I was becoming a grown-up girl. I went into an MP* [menstrual period] *for five complete years. I had no stomach pains and looked normal. But when this unusual thing happened, my parents took me to several health centres—the doctors tried all they could but they never changed the situation. In fact, one of them told my parents that I was going to run short of blood and would collapse and die there and then. When the hospitals failed to help, I was taken to other traditional healers. They gave me drinking medicine, but all the same my MP continued. As the last resort my parents brought me here to this healing centre. The healers made me sit on the mat, prayed for me for the first day, for the second day, and then for a full week—but no voice came out. But my bleeding reduced a bit. In the second week, the voice of* bachwezi *came out and said: “You have at last reached your destination. You were chosen before you were born and you are going to heal so many sick people”. Since that day, bleeding has stopped and I go into MP like other women do. When the* barangi *feel that I am healed, I will go home and start my healing place.*

(v) Grief

Eleven patients (9%) experienced the spirits of deceased persons who loved them very much; sometimes they wanted to take the patient with him/her. These experiences were often associated with grief.

Id 28. Female: age 35

The illness started after the death of my husband. Something wild came at night and held my throat so that I would even fail to breathe. My body would become so stiff. I realized it was the spirit of my dead husband. I had a lot of headache; I would long to drink cold drinks all the time. I would fall sick as if it was malaria.

The possession state was characterized by vomiting, shaking, falling down and spending up to an hour without talking/speaking or feeling anything.

My husband loved me so much. He comes often to say that to me. But the situation is very painful and unbearable.

(vi) Land conflicts

Land conflicts are a common cause of stress and possession. Sometimes this is part of the themes discussed above, such as an inheritance which has not been looked after properly, or conflicts due to jealousy of half- brothers or neighbours. Apart from these, land conflicts were mentioned in seven remaining cases.

Id 98. Female: age 37

When the healer started praying for me, I felt so excited, I started laughing, I felt so released and the headache disappeared. On the second day, the healer told me that a bad neighbour, with whom we had quarrelled over our father’s land, wanted to kill us. He had bewitched me as he had done to my sister. But she told me, so we were quick to realize it; otherwise I was going to die. I would shake whenever this healer was praying for me and sometimes I would try to speak out but the noises were not clear. I am now healed and I hope that I am about to go back home and join the rest of my family members.

(vii) Others

In some cases, spirits are mentioned without a specific cause given. These patients seemed satisfied when the spirits, who were seen as the cause of the former symptoms, were identified and subsequently disappeared after treatment. Underlying causes or conflicts did not seem to be elaborated upon in the healer’s therapy.

Id 91. Male: age 34

… Then I started falling down. Something came, it stole my mind, and it would make me fall down. Then I would shake, starting with my head and then the rest of my body, without my ability to control it. Then I was brought here to this healer, who gave me drinking medicine and some medicine to rub all around my body. I stopped falling down and shaking after some time. This healer told me it was the spirit of my late grandfather, but she assured me that it will never come back. It is now two years and, as she indeed told me, it has never come back to disturb me. I am now completely healed.

## Discussion

In this study we explored how patients with spirit possession, known to be suffering from trauma-related dissociative symptoms, evaluated their local treatment process. We explored the pathways to healing of these patients visiting traditional healers in SW Uganda by exploring their help-seeking behaviour, the healing methods used by the healers, and the generation of explanations that paralleled the healing process.

### Symptoms, help-seeking behaviour and referral

Despite the common opinion in Uganda that patients first visit traditional healers before going to a hospital [30], in our study 62% had first tried medical interventions. They were in search of a medical explanation and solution for their problems. Medical interventions, however, left their physical symptoms  medically unexplained’. In Mozambique also, spirit possession was associated with a high number of physical symptoms [12], as in Guinea Bissau [17] and in northern Uganda [14].

Western-trained doctors and health care workers often find it difficult to engage with patients with somaticized distress referred to as medically unexplained illnesses; the result is that communication stagnates. Is this an inability of emotional distressed patients to discuss their problems [43], or an inability of doctors and health workers to relate and connect to their patients’ reality? Medical workers seem to lack the necessary codes. As Leach put it: “We must know a lot of that cultural context before we can even decode its meaning” [44,45]. Medical discourse does not easily elicit cultural EMs, and health care workers usually lack the time to explore their patients’ EMs [26].

Our study demonstrates that alternative help-seeking steps were tried when medical treatment approaches were unsuccessful. In the course of this process, the signs and symptoms were  decoded’ and given meaning as expressions of spirit possession. Referral to specific healers was usually made by family members, which is consistent with the involvement of family members in other areas in Uganda [26].

### Healing type at final healing place

More than half of the healers consulted in our study were *omufumu*, traditional healers who practise traditional healing using divinition in a more or less conventional form. The *barangi* healers (30% of the consulted healers) use a mixture of traditional and Christian approaches. They have accommodated Christian beliefs and practices in their healing practices, possibly making them more acceptable to current society, which is strongly influenced by Christian morals and beliefs.

In our case group, the percentage of patients that eventually received help from religious healing at churches (17%) was relatively small. Possibly this is due to the views of the religious sector, which generally sees spirit possession as evil and as an expression of the Devil, and not as a respected spiritual messenger with relevant messages. In the Charismatic and Pentecostal churches, exorcist practices can be used to deliver one from the Devil through singing, praying, and dancing [46]. The communication attempts of possessing spirits are disposed of or suppressed, and are replaced by “the Holy Spirit”. Whereas both the church and traditional cultural approaches can be strong sources of support, conflicting approaches between the church and traditional healers can also be a strong cause of stress within families [21,26].

### Healing procedure, possessing agents, and outcome of treatment

The healing rituals narrated by the respondents showed a consistent pattern. With music, singing, and dancing, the possessing agents were invited to manifest themselves. Often the process was supported by medication or herbs, provided orally or rubbed in cuts in the body. Usually family members, community members, and other patients participated in and witnessed the happening. Possessing agents manifested themselves and expressed their anger, or warned about underlying problems. The healer negotiated with the presenting possessing agent, and the patient with his family was given advice, after which they adjusted their behaviour. This enabled the relationships with the spiritual world, families, and belongings to be restored.

Igreja described the healing process of the Gamba spirits in Mozambique as a complicated treatment process. It involved the family, who witnessed experiences of spirits of the dead appearing through the possessed patients, appearances concerning which the patient later had amnesia. Putting together the story, which formerly was fragmented and unclear, led to restored health, to morality and justice, and to reparation on the societal level [10]. The stories, however, were not always derived from the war but could originate from the former history of the family or clan. Possibly a symbolic representation of themes could also fulfil the needs. Ainemugisha, in his thesis on tradition-based approaches in transitional justice for northern Uganda, describes a detailed process of negotiation, reconciliation, and reparation used in traditional rituals and approaches, which could play a role in restoring social healing after devastating trauma and social disruption [47]. Cecil Helman in *Ritual and the Management of Misfortune*[48] describes how, in a social setting, “rituals both express and renew certain basic values of that society, especially regarding the relationships of man to man, man to nature and man to the supernatural world” (p. 225). In our study, this was well illustrated by the various examples.

Church communities can also be experienced as an important source of support in Uganda, by playing a role in facilitating collective healing and renewing social values. For example, beginning a new life as a “born-again Christian”, with “born-again brothers and sisters”, and religious rituals such as praying can assist in forgetting the (often traumatic) past. Testimonies of one’s past, given during prayer sessions, can provide an opportunity to express traumatic experiences and feelings and to leave them behind [46]. There are signs, however, that churches have become less popular owing to incidents of misbehaviour [22,26].

The fact that 99% of the patients with spirit possession report partial or full recovery after treatment is remarkable—even when taking into account that this is a selected group referred by the healers.

Unfortunately we do not have more detailed information on the time frame of outcomes and follow-ups of patients. Most of the patients were interviewed while finalizing their treatment at the healers. A few had received treatment some time before the interview and had been requested to return for the research by the healers; these were some of the patients that were now in training to become healers. Time periods between treatment and evaluation of treatment in these cases were not explicitly mentioned, and the interviewer did not probe into further details at that time. Systematic follow-up of cases was complicated as detailed address descriptions did not exist, and the availability and coverage of mobile phone connections was still limited especially in rural areas.

 Complete healing’ was reported relatively more often by patients attending the *omufumu* traditional healers and was associated with EMs related to ritual neglect and responsibilities, communicated by the *bachwezi* and ancestral spirits. EMs related to jealousy, grief, or spirits, and without a specific message, were slightly more present at the *barangi* healers and were more often related to feeling “a lot better” after treatment. Hinton and Kirmayer describe how beneficial effects of healing rituals and interventions may occur by inducing positive affective states through facilitating a shift or transformation on various levels. involving bodily experiences, affect, social interactions, and a change of the image of oneself with an increased sense of self-efficacy [49]. This may also explain the overall positive evaluation of treatment of the patients with spirit possession in our study. EMs involving ritual neglect and neglect of responsibilities provide the possibility to actively ask for forgiveness, adjust one’s behaviour, and undertake a more positive role in relation to the supra-, inter-, intra-, and extra-human world. Explanations of illness involving jealousy, grief, and “spirits without a specific reason” are less easy to solve, by their nature, and can require an attitude involving resilience and acceptance. Hinton could describe these as “a cross to bear” in a Christian religious frame. “Feeling a lot better” might be a more realistic outcome after treatment.

### Possessing agents and explanations

A considerable number of the patients with spirit possession mentioned *bachwezi* as possessing agents*.* The traditional healers described these as demi-gods, spirits of the highest category. Documentation on the *bachwezi* is scarce and often contradictory, based on oral history, myths, and legends and influenced by political views [50,51]. The discussion in internet blogs and newspaper articles shows that the debate on the origin of the *bachwezi* is receiving renewed attention [52]. In our study, the *bachwezi* spirits reminded the people of their cultural roots and lineage and urged them to revive traditional rituals and to respect connections with the past. In those patients who were called to become a healer, the *bachwezi* also played an important role.

Spirits can address taboos and conflicts and provide embodiment of simmering feelings of guilt, anger, or grief in a culture where these feelings are not easily expressed. The word “guilt” does not occur in the mini-narratives, while most of the stories are about having neglected certain responsibilities and obligations, varying from care for traditional powers and rituals, care for inheritance of family, land, or cows after someone died, or listening to signs that one should become a healer. Some patients agreed afterwards that they had done wrong and then asked for forgiveness.

Applying the model with five universal ontological dimensions mentioned by De Jong and Reis, the *bachwezi* play a strong role in restoring connections with the supra-human world and the time dimension. The ancestral spirits, the *omuzimu,* reminded people of their responsibilities towards dead and living relatives and thus appeared to have a strong role in the restoration of inter-human relationships. This can coincide with channelizing emotions in the intra-human dimension. Witchcraft seems to be more related to inter- and extra-human themes, such as jealousy of neighbours or others because of doing better business, having more land, or getting more attention.

## Conclusions

More insight into local approaches to deal with severe trauma-related dissociation can assist in developing adequate mental health services in low and middle income countries (LMIC), as well as facilitate reflection on Western psychotherapeutic approaches to dealing with trauma.

This study illustrates the need for more attention to be paid to spirit possession and dissociative disorders in mental health care and to the benefits of collaboration with traditional healers in the provision of mental health care services. Patients in SW Uganda expressed their somatoform and dissociative symptoms as medically unexplained illnesses and were often unsuccessful when seeking help in the medical sector. During the help-seeking process, these symptoms were experienced and interpreted as signs of possessing spirits, and further treatment was sought at traditional healers, Christian-influenced traditional healers, and church healing places. EMs based on spirit possession eventually led to major improvement for nearly all patients. Often-mentioned explanations were the following: neglect of spiritual rituals, neglect of responsibility towards relatives and property, the call to become a healer, witchcraft, grief, and land conflicts. Understanding of these common explanations can help to make sense of initially vague medical symptoms. The study also illustrates that traditional healers can play an important role in translating these symptoms into prevailing problems and conflicts, leading the way to solutions and healing.

The suffering and healing of patients with spirit possession was addressed in different ontological dimensions. The connection with the supra-human spiritual world and time dimension was strengthened and renewed by restoring neglected rituals and acknowledging healing powers that are passed along according to family lineages. In the inter-human dimension, ancestral sprits seemed to play a regulating role in the restoration of relationships with family and communities. In the intra-human dimension, unsettling feelings related to negligence, lack of self-restraint, or grief were addressed. The most common issue addressed in the extra-human dimension in our study were tensions about land.

EMs involving spirit possession acknowledge and address disturbed connections with the spiritual world, ancestors, family, land, and neighbours, all of which are part of one’s damaged sense of historical, cultural, and social identity. This fits with the traditional role of healers, that of maintaining and restoring balance in the community and with the spiritual world. These issues are easily overlooked in Western trauma therapy. Treatment of dissociative identity disorders in Western countries, commonly attributed to severe and chronic childhood traumatization, emphasizes integrating individual traumatic experiences within a phase-oriented therapy. This often resulted in the difficulty of therapists and patients getting lost in lengthy therapies. When it was acknowledged that the focus on traumatic memories was insufficient, phase-oriented therapy was adjusted to provide more attention to attachment and social integration [35,53]. As De Jong and Reis illustrate, the emphasis of Western therapy is mostly on the intra-human dimension, with limited attention given to the inter-human and time dimensions. Supra-human and extra-human dimensions rarely play a role in Western therapy. The results of this study suggest that involving a spiritual, religious, and social perspective can perhaps make therapy for patients with trauma-related dissociation more effective.

The study can also contribute to the debate on effective mental health policies and systems for underserved populations. Our research in Uganda, as well as the other mentioned research on dissociation in African and other low-income countries, demonstrates the importance of strengthening social and spiritual resources that can lead to social cohesion and enable healing. Knowledge of cultural themes and explanations and a systemic approach can be helpful in dealing with trauma-related dissociative symptoms. There is also some evidence that uncovering and unravelling individual traumatic experiences is not always necessary to increase mental well-being. It is possible that restoration of social, cultural, and spiritual belonging provides strength to endure certain individual traumatic experiences. At the same time, cultural themes can also allow patients to process trauma on a symbolic level. Verbalizing individual stresses and traumas in psychological EM can be dangerous within certain social, cultural, or political contexts [17,54]. Sometimes, however, when individual traumatic experiences and therapeutic needs may be insufficiently addressed with a cultural–spiritual approach (which may be perceived as an anachronism), individual counselling and psychosocial support interventions can also play an important role [55,56]. When explanations—whether medical, psychological, social, political, cultural, or spiritual–religious—do not seem to lead to a solution and are experienced as  paralyzing’, a shift of EM can lead to another dimension with new perspectives and solutions and result in an increased sense of patient self-efficacy.

## Endnotes

^a^The burning of items, which had been used for traditional rituals was witnessed by the first author at a Charismatic Church gathering in Rubindi, in 2000.

^b^The *emandwa* cults have been described as occurring in Ankole and Rwanda. The *emandwa* are described as benevolent guardian spirits of lineage groups (*ekibunu*), of which at least one member must always be initiated into the cult [50]. In meetings with traditional healers, I was told *emandwa* spirits are messenger spirits and in hierarchy are between the *bachwezi* and ancestral spirits.

^c^Dreams about white men and a corral of cattle probably refer to the *bachwezi*. They were seen as demi-gods or godly powers in charge of a high civilization that later vanished. They are described as tall, light-skinned men who were pastoralists, herding long-horned cattle, and originally came from Egypt. Others are of the opinion the light skins of the *bachwezi* is an invention of former colonial rulers. Also, the lineage connection to these representatives of a high civilization claimed by certain clans is seen by others as being used for political gain, power, and “proof of superiority” [52].

## Competing interests

The authors declare that they have no competing interests.

## Authors’ contributions

MvD carried out the research in Uganda and drafted the manuscript. WK participated in the design of the study and contributed to the statistical analysis. JdJ participated in the design and coordination of the study and helped to draft the manuscript. All authors read and approved the final manuscript.
